# International consensus on post-transplantation diabetes mellitus

**DOI:** 10.1093/ndt/gfad258

**Published:** 2024-01-03

**Authors:** Adnan Sharif, Harini Chakkera, Aiko P J de Vries, Kathrin Eller, Martina Guthoff, Maria C Haller, Mads Hornum, Espen Nordheim, Alexandra Kautzky-Willer, Michael Krebs, Aleksandra Kukla, Amelie Kurnikowski, Elisabeth Schwaiger, Nuria Montero, Julio Pascual, Trond G Jenssen, Esteban Porrini, Manfred Hecking

**Affiliations:** Department of Nephrology and Transplantation, University Hospitals Birmingham, Birmingham, United Kingdom; Institute of Immunology and Immunotherapy, University of Birmingham, Birmingham, United Kingdom; Division of Nephrology and Hypertension, Mayo Clinic, Scottsdale, AZ, United States of America; Leiden Transplant Center, Leiden University Medical Center, Leiden, The Netherlands; Department of Nephrology, Leiden University Medical Center, Leiden, The Netherlands; Division of Nephrology, Department of Internal Medicine, Medical University of Graz, Graz Austria; Department of Diabetology, Endocrinology, Nephrology, University of Tübingen, Tübingen, Germany; Ordensklinikum Linz, Elisabethinen Hospital, Department of Medicine III, Nephrology, Hypertension, Transplantation, Rheumatology, Geriatrics, Linz, Austria; Medical University of Vienna, CeMSIIS, Section for Clinical Biometrics, Vienna, Austria; Department of Nephrology, Copenhagen University Hospital Rigshospitalet, Copenhagen, Denmark; Department of Transplantation Medicine, Oslo University Hospital, Rikshospitalet, Oslo, Nydalen, Norway; Department of Nephrology, Oslo University Hospital-Ullevål, Oslo, Nydalen, Norway; Department of Internal Medicine III, Clinical Division of Endocrinology and Metabolism, Medical University of Vienna, Vienna, Austria; Department of Internal Medicine III, Clinical Division of Endocrinology and Metabolism, Medical University of Vienna, Vienna, Austria; Division of Nephrology and Hypertension, Mayo Clinic, Rochester, MN, United States of America; William J. von Liebig Center for Transplantation and Clinical Regeneration, Mayo Clinic, Rochester, MN, United States of America; Department of Internal Medicine III, Clinical Division of Nephrology and Dialysis, Medical University of Vienna, Vienna, Austria; Department of Internal Medicine, Brothers of Saint John of God Eisenstadt, Eisenstadt, Austria; Nephrology Department, Hospital Universitari de Bellvitge, L’Hospitalet de Llobregat, Barcelona, Spain Biomedical Research Institute (IDIBELL), L’Hospitalet de Llobregat, University of Barcelona, Barcelona Spain; Institute Mar for Medical Research-IMIM, Barcelona, Spain; Department of Nephrology, Hospital Universitario 12 de Octubre, Madrid, Spain; Department of Transplantation Medicine, Oslo University Hospital, Rikshospitalet, Oslo, Nydalen, Norway; Institute of Clinical Medicine, University of Oslo, Oslo, Norway; Instituto de Tecnologías Biomédicas (ITB), University of La Laguna, Research Unit Department, Hospital Universitario de Canarias, Tenerife, Spain; Department of Internal Medicine III, Clinical Division of Nephrology and Dialysis, Medical University of Vienna, Vienna, Austria; Center for Public Health, Department of Epidemiology, Medical University of Vienna, Vienna, Austria; Kuratorium for Dialysis and Kidney Transplantation (KfH), Neu-Isenburg, Germany

**Keywords:** GLP-1 analogues, metabolic syndrome, NODAT, post-transplant diabetes mellitus, SGLT2 inhibitors

## Abstract

Post-transplantation diabetes mellitus (PTDM) remains a leading complication after solid organ transplantation. Previous international PTDM consensus meetings in 2003 and 2013 provided standardized frameworks to reduce heterogeneity in diagnosis, risk stratification and management. However, the last decade has seen significant advancements in our PTDM knowledge complemented by rapidly changing treatment algorithms for management of diabetes in the general population. In view of these developments, and to ensure reduced variation in clinical practice, a 3rd international PTDM Consensus Meeting was planned and held from 6–8 May 2022 in Vienna, Austria involving global delegates with PTDM expertise to update the previous reports. This update includes opinion statements concerning optimal diagnostic tools, recognition of prediabetes (impaired fasting glucose and/or impaired glucose tolerance), new mechanistic insights, immunosuppression modification, evidence-based strategies to prevent PTDM, treatment hierarchy for incorporating novel glucose-lowering agents and suggestions for the future direction of PTDM research to address unmet needs. Due to the paucity of good quality evidence, consensus meeting participants agreed that making GRADE (Grading of Recommendations, Assessment, Development, and Evaluations) recommendations would be flawed. Although kidney-allograft centric, we suggest that these opinion statements can be appraised by the transplantation community for implementation across different solid organ transplant cohorts. Acknowledging the paucity of published literature, this report reflects consensus expert opinion. Attaining evidence is desirable to ensure establishment of optimized care for any solid organ transplant recipient at risk of, or who develops, PTDM as we strive to improve long-term outcomes.

## INTRODUCTION

Post-transplantation diabetes mellitus (PTDM) significantly contributes to morbidity and mortality after solid organ transplantation (SOT). The last International PTDM Consensus Meeting in 2013 consolidated heterogenous clinical practice and suggested standards of care for the screening, diagnosis and management of PTDM [1]. However, the PTDM field has evolved dramatically since 2013, justifying an update. Research has enhanced our understanding, while expanded therapeutic options in the general population have dramatically shifted treatment algorithms. In this rapidly changing climate, ambitions to improve long-term SOT outcomes require optimized strategies to prevent/manage PTDM that are aligned with the latest scientific updates.

This Meeting Report summarizes proceedings from the 3rd International PTDM Consensus Meeting held in Vienna, Austria, from 6–8 May 2022. The meeting was endorsed by the European Renal Association (Diabesity Working Group) and the European Society for Organ Transplantation (EKITA Working Group). An international expert panel was convened by invitation, comprising 18 transplant clinicians, diabetologists and scientists with an active interest in the field, to deliberate updates to the previous consensus statement relevant for contemporary clinical practice. Invitations were based upon a meeting prerequisite to systematically review existing literature for presentation at open scientific sessions, encouraging debate and discussion. While targeting all SOT recipients, published data are kidney-centric and organ-specific considerations are required. After reviewing and reflecting upon the paucity of good quality evidence, consensus opinion agreed that making GRADE (Grading of Recommendations, Assessment, Development, and Evaluations) recommendations would be flawed [2]. Therefore, our terminological use of ‘Opinion Statement’ is deliberate to acknowledge this. This position statement reflects the consensus view of expert delegates. Ultimately, attaining this evidence is desirable to ensure establishment of optimized care for any solid organ transplant recipient at risk of, or who develops, PTDM as we strive to improve long-term outcomes.

## OPINION STATEMENT 1: PERFORM AN ORAL GLUCOSE TOLERANCE TEST FOR DIAGNOSIS AND SCREENING; START ON THE WAITING LIST

Glucose thresholds for defining diabetes in the general population are based on the probability of developing retinopathy [3], but only one study explores this issue post-transplantation [4]. An oral glucose tolerance test (OGTT) is essential for diagnosis and screening (see Supplementary data, Table S1), as alternatives like haemoglobin A1c (HbA1c) lack diagnostic sensitivity [5–7] and association with adverse outcomes [1, 8, 9]. Patients with impaired glucose tolerance (IGT), exclusively diagnosed by OGTT, or PTDM are at risk for cardiovascular disease [9] and premature death [1, 8]. Importantly, OGTTs allow earlier identification of at-risk individuals on the waiting list [10]. When diagnosed early or by 2-h postprandial glucose only, PTDM may have greater chance of reversibility, although this may reflect low reproducibility [11]. Supplementary data, Table S2 summarizes the published evidence.

**Figure 1:**
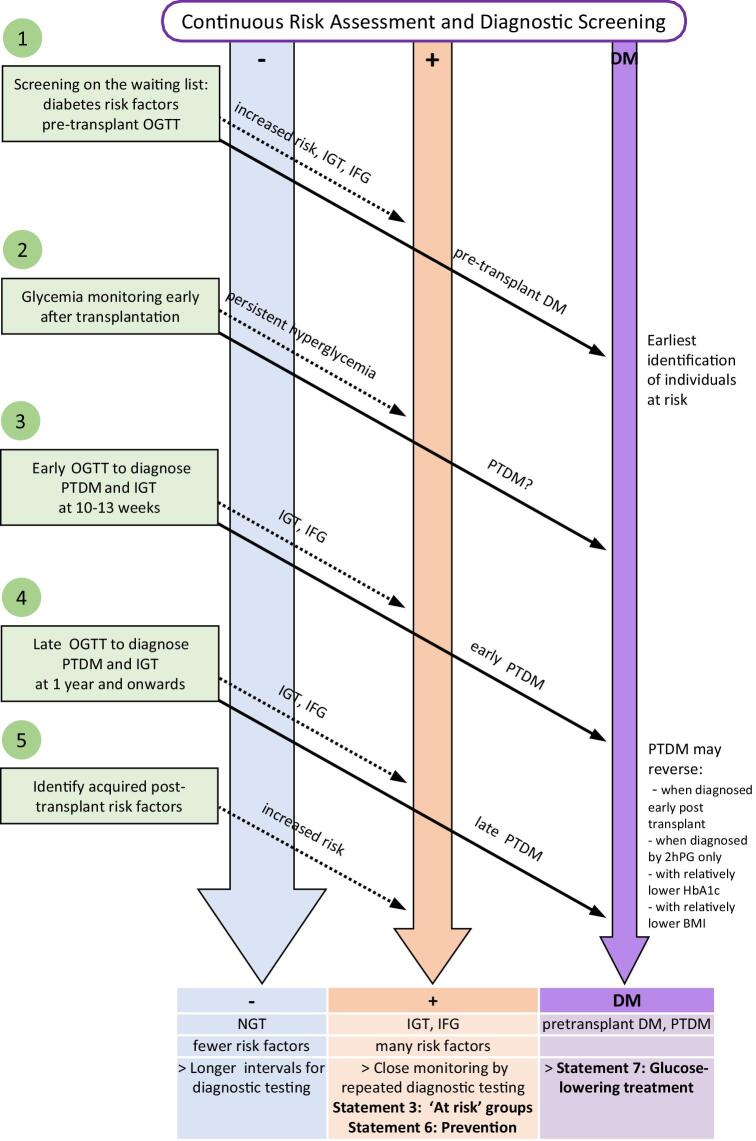
Five aspects of risk assessment for and diagnosis of PTDM and IGT.

Long-term evolution of PTDM is characterized by metabolic variability [7, 11, 12]. Individuals with prediabetes (impaired fasting glucose and/or IGT) or PTDM risk factors will benefit from repeated (e.g. annual) OGTT testing. If diagnosed early (e.g. 3 months post-operatively), PTDM may need later confirmation. A diagnosis and screening algorithm is proposed (Fig. 1) but warrants validation for improvement of outcomes.

## OPINION STATEMENT 2: BE AWARE OF LONG-TERM CONSEQUENCES OF PREDIABETES AND PTDM

PTDM is associated with overall graft loss [13], cardiovascular events [8, 14] and all-cause mortality [8], while microvascular complications are less studied [4] and patient-reported outcomes are scarce. Some studies observe no association with patient/graft survival [15, 16], but this discrepancy might be influenced by heterogenous cohorts, diagnostic criteria or methodological differences. Importantly, the association of prediabetes with mortality and cardiovascular events should be appreciated [9]. Other long-term consequences of PTDM require evaluation. For example, diabetes is associated with several cancers in the general population but data with PTDM are limited. A recent cohort analysis has observed an association between PTDM and future renal cell cancer [17], consistent with observations from a Danish cohort describing increased risk for cardiovascular and cancer-related mortality in SOT recipients with pre-transplant diabetes or PTDM [18].

## OPINION STATEMENT 3: PRIORITIZE CLINICAL ATTENTION TO ‘AT RISK’ GROUPS

SOT recipients are at risk for the development of prediabetes/PTDM, but certain patients have a disproportionately higher risk. Early identification of this high-risk group is crucial to ensure that resources are directed to the most vulnerable, who may be amenable to intervention.

This ‘at-risk’ group can be classified by clinical phenotypes or novel risk prediction methods like polygenic risk scores (PRS). The latter estimates an individual's genetic liability for a specific disease according to their genotypic profile and has been studied after liver and kidney transplantation [19]. PRS are associated with pre-transplant type 2 diabetes and post-surgery PTDM. PRS in liver donors, but not kidney donors, was an independent risk factor for PTDM development and a combined liver donor/recipient PRS improved PTDM prediction over-and-above a clinical variable model alone. Further research is recommended to identify the optimal way to identify at-risk groups.

## OPINION STATEMENT 4: CONSIDER UNDERLYING PATHOMECHANISM OF PTDM DEVELOPMENT AND THE INTER-RELATIONSHIP BETWEEN β-CELL DYSFUNCTION AND METABOLIC STRESS

PTDM arises from an interaction between pre-transplant and post-transplant risk factors (Supplementary data, Fig. S1). Many pre-transplant risk factors are common to type 2 diabetes (i.e. obesity, metabolic syndrome), but immunosuppression is the most important post-transplant risk factor. Pre-transplant risk factors may identify individuals at risk from immunosuppression-induced β-cell toxicity amenable to intervention, supporting the use of waiting-list screening.

Mechanistically a combination of pancreatic β-cell dysfunction and insulin resistance are predisposing factors for PTDM, with superimposed immunosuppression accelerating pre-existing damage [20]. A mechanistic approach is depicted in Supplementary data, Fig. S2 according to an animal model of calcineurin inhibitor (CNI)-induced toxicity, potentiating similar cellular damage induced by obesity and insulin resistance, which indicates common pathways in β-cell dysfunction [20]. Importantly, this principle has been corroborated with slightly different pathways in human islets and pancreas transplant biopsies [21]. Tacrolimus induces β-cell damage provoked by the glucolipotoxicity state secondary to multi-factorial insults, pathogenic pathways [e.g. mammalian target of rapamycin (mTOR) pathway] [22] responsible for β-cell maintenance and function [20]. Furthermore, low-grade inflammatory stress is associated with early occurrence of PTDM [23] and early post-transplant mortality in general [24]. Thus, a ‘two-hit’ hypothesis combining transplantation-induced β-cell insult on a background of metabolic stress converging in a dysfunctional synergy is an attractive hypothesis for the development of prediabetes/PTDM. However, other confounders must not be overlooked. For example, Halden *et al*. demonstrated infusion of the incretin hormone glucagon-like peptide 1 (GLP-1) during fasting and hyperglycaemic conditions in patients with PTDM compared with normal glucose tolerance, rectified pathophysiological defects like hyperglucagonemia, and diminished first- and second-phase insulin secretion [25].

## OPINION STATEMENT 5: CHOOSE AN IMMUNOSUPPRESSION REGIMEN FOR OPTIMIZATION OF PATIENT AND GRAFT SURVIVAL

Despite the association between immunosuppression and PTDM, *de novo* regimens should not be routinely modified to reduce PTDM risk or adjusted after PTDM development. However, for selected patients, tailored immunosuppression may be justified if development of diabetes outweighs other risks. Patient-specific factors, immunological considerations and competing risks must all be factored when choosing immunosuppression on a personalized basis.

No robust data link induction therapy directly to PTDM risk. However, lymphocyte-depletion therapies (e.g. thymoglobulin, alemtuzumab) can facilitate lower exposure to maintenance CNIs and steroids which can reduce PTDM risk.

Regarding CNIs, Torres *et al*. randomized 128 *de novo* kidney transplant recipients (KTRs) at high-risk for PTDM but low immunological risk to: (i) tacrolimus and rapid steroid withdrawal, (ii) cyclosporine and steroid maintenance, or (iii) tacrolimus with steroid maintenance [26]. All arms received basiliximab and steroids. Patient/graft survival and graft function were similar between study arms, with tacrolimus and steroid maintenance providing the best balance between risk for PTDM versus acute rejection. There is limited evidence supporting conversion of CNI in established PTDM. In a randomized controlled trial (RCT) involving 87 KTRs, conversion from tacrolimus to cyclosporine significantly improved glycaemic control with no increased risk for acute rejection [27]. Late changes to immunosuppressive regimens may alleviate PTDM but this requires further evaluation to ensure glycaemic benefits outweigh long-term allograft risks. There is not enough evidence to support using different tacrolimus formulations, such as immediate versus prolonged release, but results from ongoing studies are awaited (see Supplementary data, Table S3).

Belatacept has a favourable metabolic risk profile, including less PTDM [28], in comparison with CNIs and different regimens have been explored in RCTs including KTRs [29]. Belatacept is an acceptable alternative to CNIs to reduce PTDM in low immunological–risk patients if logistical and cost implications are surmountable. Any studies to explore efficacy in non-renal SOT recipients should ensure data capture of PTDM as a secondary outcome.

Although mTOR inhibitors are diabetogenic, incidence of PTDM is not significantly increased by their use which may reflect reduced CNI exposure. A recent meta-analysis evaluating the combination of CNI plus mTOR inhibitors in *de novo* KTRs observed no increase of 1-year PTDM versus CNI plus antiproliferative agents in 13 studies [*n* = 4561 participants; relative risk 1.16, 95% confidence interval (CI) 0.97–1.38, *P *= .10] [30]. These results were confirmed in the TRANSFORM (TRANSplant eFficacy and safety Outcomes with an eveRolimus-based regiMen) study, a 24-month, prospective, open-label trial in 2037 *de novo* KTRs randomized to receive everolimus with reduced-exposure CNI versus mycophenolate with standard-exposure CNI [31]. No difference in PTDM incidence was observed (risk ratio 1.09, 95% CI 0.87–1.37) with comparable efficacy and graft function.

There is no evidence to suggest any glycaemic risk from anti-proliferative agents such as mycophenolate mofetil or azathioprine.

Regarding steroids, a previous Cochrane analysis published in 2016 observed similar rates of mortality, graft loss and PTDM comparing regimens of steroid avoidance/withdrawal (stratified before or after 14 days, respectively) versus steroid maintenance, but higher rates of rejection [32]. In an updated analysis incorporating post-2016 RCTs of steroid avoidance [33, 34], lower rates of PTDM are now observed in steroid avoidance versus maintenance (risk ratio 0.70, 95% CI 0.56–0.88, *P *= .002) but with similar mortality, graft loss and rejection observations to before (see Supplementary data, Fig. S3). However, the HARMONY study contributes a large effect size but is flawed by overreliance on HbA1c for PTDM diagnosis in the context of anemia rates between 27% and 39% across study arms [33]. Early steroid withdrawal may have differential impact stratified by age, with older SOT recipients in a population-cohort study demonstrating more favourable responses to steroid withdrawal (e.g. lower PTDM and mortality) but increased risk for rejection [35]. Balancing PTDM versus graft-related concerns with steroid avoidance/withdrawal is essential, although patient/graft survival should take priority. In a causal estimation effects registry analysis including 6070 KTRs, steroid withdrawal within 18 months post-transplantation was associated with increased risk of graft loss compared with steroid maintenance [36]. If a steroid avoidance regimen is desired then induction therapy with lymphocyte depletion should be considered.

## OPINION STATEMENT 6: EMPHASIZE LIFESTYLE MODIFICATION TO ALL PATIENTS; CONSIDER MEDICAL OR SURGICAL INTERVENTION FOR TREATMENT OF OBESITY; USE INTERMITTENT EXOGENOUS INSULIN INTERVENTION EARLY POST-TRANSPLANTATION FOR POST-OPERATIVE HYPERGLYCAEMIA

Since the last meeting report [1], various groups have summarized suggestions on PTDM prevention [37–41]. These include: (i) dietary modification; (ii) physical exercise/training; (iii) pharmacological intervention; (iv) immunosuppression modification; (v) bariatric surgery; (vi) performing OGTTs pre-transplant for targeted intervention; and (vii) other measures including manipulation of microbiota. Meeting participants agreed any opinion regarding prevention would intuitively become stronger with increasing PTDM risk.

Regarding (i), uncertainty exists about the best dietary intervention [42], as observational evidence only supports Mediterranean diets [43] or increased vegetable intake [44]. With (ii), the CAVIAR (Comparing glycaemic benefits of Active Versus passive lifestyle Intervention in kidney Allograft Recipients) RCT implemented a graded exercise program with active dietician intervention (versus leaflet advice), which did not improve pathophysiological markers of glucose metabolism but reduced PTDM incidence [45]. An observational study demonstrated higher physical activity levels lowered risk of PTDM, and cardiovascular and all-cause mortality [46]. Although better evidence is desirable, meeting participants agreed that lifestyle modification, combining measures (i) and (ii), should be emphasized post-transplantation based upon evidence from the general population [47].

As for (iii), meeting participants agreed early exogenous insulin administration could be considered for PTDM prevention despite a recent RCT not reaching its primary endpoint (1-year PTDM incidence) [48]. This agreement acknowledged that the odds for overt PTDM at 1-year were significantly reduce in the adjusted per-protocol analysis only [48], and was also based on an earlier RCT (cited in previous meeting report) [1]. However, higher hypoglycaemia rates with this approach must be acknowledged [48] and enthusiasm may be influenced by inpatient length of stay post-operatively. An ongoing multicentre RCT testing early administration of vildagliptin for PTDM prevention is underway (Supplementary data, Table S3) [49], but another RCT was recently published demonstrating that post-operative sitagliptin was safe but did not lead to significant improvement in OGTT-derived 2-h glucose at 3 and 6 months post-transplantation [50].

The most controversial issue with PTDM prevention is immunosuppression tailoring for SOT patients at higher PTDM risk as per (iv), which is addressed under Opinion Statement 5. Meeting participants agreed further research is warranted to investigate immunosuppression modification strategies to prevent or treat PTDM.

Concerning (v), there is convincing evidence that bariatric surgery is beneficial for individuals with morbid obesity and chronic kidney disease (CKD), including those already waitlisted or seeking eligibility [51, 52]. In kidney transplant candidates with obesity (e.g. body mass index ≥35 kg/m^2^) refractory to lifestyle intervention, consider surgical or medical intervention which will enable successful transplantation and may aid PTDM prevention. A non-randomized study reported zero cases of PTDM in 12 non-diabetic KTRs transplanted after post-laparoscopic sleeve gastrectomy, in comparison with 3 of 18 patients from a matched non-laparoscopic sleeve gastrectomy control group (statistically not significant) [53]. As an alternative, GLP-1 receptor agonists might be a promising pharmacological option for individuals with advanced CKD and obesity who are transplant candidates. Studies are pending to determine feasibility (Supplementary data, Table S3).

Regarding measures (vi) and (vii), Hap *et al*. performed OGTTs among 80 waitlisted kidney transplant candidates and recommended a low carbohydrate diet, lifestyle modification and increased physical activity to 31 patients with dysglycaemia (with 28/31 showing attenuated glucose metabolism throughout the 12-month observational period post-transplant) [54]. These results align with several measures highlighted above showing that behavioural factors such as motivation are important to enable PTDM prevention.

## OPINION STATEMENT 7: USE THE NOVEL AGENTS; PERSONALIZE GLUCOSE-LOWERING THERAPY BASED UPON A PATIENT-DEPENDENT HIERARCHY

**Figure 2:**
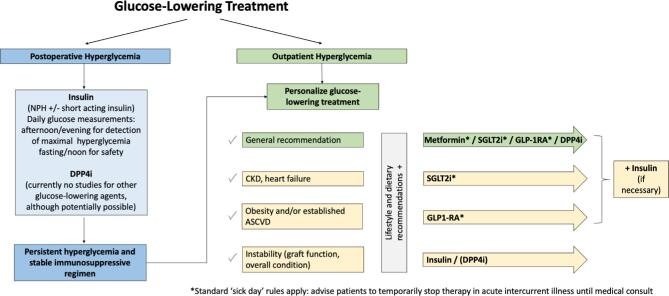
Glucose-lowering treatment in KTRs: suggested algorithm.

Cardiovascular outcome trials using glucose-lowering treatment in KTRs are lacking. Novel agents, sodium-glucose co-transporter 2 (SGLT2) inhibitors and GLP-1 receptor agonists, now dominate diabetes treatment guidelines [55]. Meeting participants agreed that novel agents are under-utilized for PTDM management due to limitations of transplant-specific evidence (see Tables 1A/1B). However, prescribing is sub-optimal even in diabetic kidney disease patients in whom there are clear treatment benefits as per national/international recommendations [56]. This reflects a disconnect between clinical guidelines and real-world prescribing. Available transplant studies do not currently indicate a clear safety risk, which is why our personal view is more enthusiastic in comparison with recent KDIGO guidance on diabetes and CKD recommending more cautious adoption [57]. Meeting participants agreed targeted PTDM studies are desirable but adoption should not be delayed based on current evidence. Meeting participants also agreed that initiation of glucose-lowering agents will be reliant upon accessibility. However, if accessibility is not an issue, then a patient-dependent hierarchy (Fig. 2) is advisable.

**Table 1A:** Prospective studies on glucose-lowering agents after kidney transplantation.

**Study**	**Study size and design**	**Duration**	**Intervention/comparator**	**Primary outcome/main outcome**	**Secondary outcomes**	**Primary outcome results/outcome results**	**Strength**	**Weakness**
Insulin								
Hecking *et al*. 2012 [65]	*N* = 56; RCT	12 months	Basal (NPH) insulin ± short acting insulin/standard of care	Difference in HbA1c at Month 3	Difference in HbA1c at Month 6 and 12, prevalence of NODAT and IGT, capillary blood glucose profile and the amount of insulin needed	HbA1c at 3 months was significantly different and lower in the basal insulin group	First study to prove that PTDM might be preventable. Pathophysiologically plausible	Patients who dropped out were replaced. Small single-centre analysis. Results presented as unadjusted and adjusted results
Schwaiger *et al*. 2021 [48]	*N* = 263; RCT	24 months	Basal (NPH) insulin ± short acting insulin/standard of care	PTDM at 1 year	PTDM at 2 years, glycaemic control, kidney function, patient and graft survival	PTDM risk in unadjusted and adjusted ITT analyses: no statistically significant difference was observed between groups; PTDM risk in unadjusted PP analysis: no statistically significant difference was observed between groups; PTDM risk in adjusted PP analysis: a statistically significant difference with lower occurrence of PTDM was observed in the basal insulin group	Relatively large multicentre RCT compared with other PTDM studies	Significant baseline differences regarding ADPKD. Protocol deviations as described by the authors
Metformin								
Alnasrallah *et al*. 2019 [66]	*N* = 19; pilot RCT	3–12 months	Metformin/standard of care (lifestyle instruction)	Feasibility of recruitment, tolerability of metformin, efficacy of metformin in IGT	Lipid profile, change in body weight, cardiac events, adverse events, proportion of patients who revert to normal glucose metabolism, drug discontinuation, SAE	19 patients out of 78 with an OGTT recruited. Tolerability of metformin comparable between groups at 3 and 12 months. Efficacy of metformin on HbA1c and fasting plasma glucose not different at the tested time points	First RCT with metformin in transplanted patients, focus on prevention (IGT patients), patient education taken seriously	Sample size too small to prove absence of lactic acidosis
Thiazolidinediones								
Baldwin and Duffin 2004 [67]	*N* = 18 (*N* = 11 with DM2, *N* = 7 with PTDM); prospective, observational (interventional)	133–718 days	Rosiglitazone	HbA1c improvement, avoidance of PTDM, avoidance of insulin dependency in PTDM	Blood levels of cyclosporin A, tacrolimus, creatinine. Weight, peripheral oedema, pulmonary congestion, liver enzyme, lipids	HbA1c improved significantly in DM2 and PTDM, PTDM patients did not depend on insulin	Novelty at that time. Duration of follow-up	Study design itself, potential selection bias, sample size
Villanueva *et al*. 2005 [68]	*N* = 40; prospective, observational (interventional)	12 months	Rosiglitazone	To evaluate the effect of rosiglitazone on insulin resistance in PTDM	Physical examination, serum chemistry, weight, cyclosporin and tacrolimus levels	91% of patients initially treated with insulin were able to discontinue insulin. 30% were controlled with rosiglitazone monotherapy. Serum creatinine was stable during treatment with rosiglitazone. 13% treated with rosiglitazone developed oedema	Real-world study	Immunosuppressive regimen was modified
Voytovich *et al*. 2005 [69]	*N* = 10; prospective, observational (interventional)	4 weeks	Rosiglitazone	Impact on insulin sensitivity, plasma glucose and endothelial function in KTR with glucose intolerance	Safety parameters	Mean glucose disposal rate increased, the mean fasting plasma glucose and 2-h plasma glucose fell significantly, AUC glucose (from OGTT) was sig. reduced. Insulin secretion was not reduced. No sig. association between lowering plasma glucose and the improvement of endothelial function	Mechanistically sophisticated (clamp-derived measurement of insulin sensitivity), pathophysiologically insightful	Relatively short treatment time which limits clinical interpretation (also of safety parameters)
Han *et al*. 2010 [70]	*N* = 83; RCT	12 months	Pioglitazone/control (not receiving pioglitazone)	Mean and max. carotid IMT	Adiponectin levels, lipids, insulin secretory function and sensitivity	Mean max IMT decreased only in the pioglitazone group. Association of adiponectin and IMT in pioglitazone group. Pioglitazone increased insulin sensitivity	Study design, sample size, endpoints not limited to glucose metabolism	Not placebo-controlled
Werzowa *et al*. 2013 [71]	*N* = 52; RCT	3 months	Vildaglitpin/pioglitazone/placebo	Difference in change in OGTT-derived 2-h plasma glucose	Difference in 2hPG, FPG, HbA1c and fasting insulin within the groups before and after treatment, change in kidney and liver function, side effects	The primary endpoint did not reach statistical significance	Diabetologically comprehensive. The only study in prediabetes	Weak effects. Limited pathophysiological information
Meglitinides								
Voytovich *et al*. 2007 [72]	*N* = 14 (*N* = 6 with PTDM, *N* = 8 with IGT); prospective, observational (interventional)	2 weeks	Nateglinide	Insulin response and glucose excursions after a standardized liquid meal	Carbohydrate and fat oxidation rates in indirect calorimetry, insulin, C-peptide, free fatty acids, triglycerides, lipids, liver enzymes, and creatinine, CsA and tacrolimus levels	Significant decrease in 2hPG, decline of AUC _glucose0–240 min_, increase of AUC _ins0–30 min_, AUC _ins30–120 min_, and AUC_C-peptide_. Lower postprandial glucose in self-measurements	Proof of mechanism of action	Relatively short treatment time which limits clinical interpretation (also of safety parameters)
GLP-1 receptor agonists								
Pinelli *et al*. 2013 [73]	*N* = 5; prospective, observational (interventional) (case series)	3 weeks	Liraglutide	Tacrolimus AUC_0–12h_	Tacrolimus trough levels, allograft function, blood glucose	Tac-AUC reduced, Tac trough levels unaltered, reduction of postprandial glucose and body weight	The only study reporting an AUC for tacrolimus under GLP-1-RA treatment	Small sample size, descriptive, difference in Tac-AUC not emphasized in the conclusion
Halden *et al*. 2016 [25]	*N* = 24; RCT	2–4 weeks	GLP-1 infusion/0.9% saline. Hyperglycaemic clamp	Fasting levels of plasma glucose, glucagon, and insulin, AUC concentrations	Glucagon, proinsulin and insulin secretory response to arginine	Patients with PTDM showed a reduced ability to suppress circulating glucagon levels during the hyperglycaemic clamp. First- and second-phase insulin secretion was lower compared with the control group	Pathomechanistically sophisticated	Relatively short treatment time which limits clinical interpretation (also of safety parameters)
DPP4 inhibitors								
Lane *et al*. 2011 [74]	*N* = 15; prospective, observational (interventional) (pilot study)	3 months	Sitagliptin	Effect of sitagliptin on tacrolimus and sirolimus levels and changes in renal function	Side effects and change in HbA1c	Significant reduction in HbA1c, no significant change in tacrolimus or sirolimus levels, no significant change in eGFR	First report on DPP4 inhibitors in transplanted patients	None, apart from small sample size and descriptive design
Werzowa *et al*. 2013 [71]	*N* = 52; RCT	3 months	Vildaglitpin/pioglitazone/placebo	Difference in change in 2hPG	Difference in 2hPG, FPG, HbA1c and fasting insulin within the groups before and after treatment, change in kidney and liver function, side effects	The primary endpoint did not reach statistical significance	Diabetologically comprehensive. The only study in prediabetes	Weak effects. Limited pathophysiological information
Soliman *et al*. 2013 [75]	*N* = 62; RCT	12 weeks	Sitagliptin/insulin glargine	Change in HbA1c from baseline to Week 12	Change in body weight, fasting plasma glucose, lipid profile	Significant reduction in HbA1c and fasting plasma glucose, comparable to insulin	Study design clinically meaningful, answering a clinical need at that time	Many drop-outs in the insulin group
Haidinger *et al*. 2014 [76]	*N* = 33; RCT	3 months (active), 4 months (including follow-up)	Vildaglitpitn/placebo	Difference in the intraindividual change in OGTT-derived 2hPG between	Differences between the intraindividual change in OGTT-derived 2hPG from baseline to 4 months, FPG, HbA1c and fasting insulin, rate of side-effects, change in eGFR, albuminuria/proteinuria, change in liver function parameters from baseline, and immunosuppressant serum levels	Intraindividual change in 2hPG between the vildagliptin, and placebo group was statistically significant at Month 3	OGTTs with insulin sensitivity and secretion during treatment and 1 month after drug discontinuation	Short treatment duration
Strøm Halden *et al*. 2014 [77]	*N* = 19; cross-over RCT	8 weeks (4 weeks treatment)	Sitagliptin/sitagliptin-free	Effect of sitagliptin on insulin secretion	Plasma glucose, insulin sensitivity, endothelial function, safety parameters (calcineurin inhibitor/everolimus levels and changes in renal function)	Median (IQR) first- and second-phase insulin secretion responses increased following sitagliptin treatment as compared with control	Study clinically well-intended to ensure treatment in all patients, OGTTs with insulin sensitivity and secretion, markers on cardiovascular risk	Patients had different CNIs, temporal effects due to cross-over design and relatively short treatment duration
Delos Santos *et al*. 2023 [50]	*N* = 61; RCT	6 months	Sitagliptin/placebo	OGTT-derived 2-h glucose at 3 months	PTDM prevention at 3 months (defined by normal OGTT)	OGTT-derived 2-h glucose was 24 mg/dL lower and PTDM risk reduction was 18% in the sitagliptin group (not significantly different)	Mechanistically compelling. The first study on PTDM prevention to date using a DPP4 inhibitor	As lower 2-h glucose among patients on treatment was expectable, more information could have been derived and presented from the OGTTs
SGLT2is								
Schwaiger *et al*. 2019 [78]	*N* = 14, *N* = 24 matched reference patients with PTDM; prospective, observational (interventional)	4 weeks run in, 4 weeks empagliflozin monotherapy, 12 months follow-up	Empagliflozin monotherapy, followed by empagliflozin as add on	Intra‐individual difference in the 2hPG between the baseline OGTT and the OGTT after 4 weeks: non-inferiority design	Laboratory parameters, anthropometric measurements, blood pressure, and medications. Bioimpedance spectroscopy‐based assessment of fluid volume status and body composition, urinary tract infections compared with reference group	OGTT-derived 2hPG increased during 4 weeks of empagliflozin treatment (*P* = ns), demonstrating clinically inferiority	Many endpoints studied	Inferiority of empagliflozin as substitute for insulin would have been expectable, small sample size
Halden *et al*. 2019 [62]	*N* = 44; double-blind RCT	24 weeks	Empagliflozin/placebo	Change in weighted mean glucose estimated with continuous glucose monitoring from iPro2	Change in HbA1c, FPG, 2hPG in OGTT, body weight, WHR, body composition including visceral fat, blood pressure, and eGFR	Primary endpoint not evaluated (technical error), median change in HbA1c significantly reduced after 24 weeks of empagliflozin treatment compared with placebo	Sophisticated study design, the authors confirmed that SGLT2is have no glucose-lowering effect at eGFR <45 mL/min/1.73 m^2^ in transplanted patients	Prespecified primary endpoint not analysed, some uncertainty remains regarding urinary tract infections
Mahling *et al*. 2019 [79]	*N* = 10; prospective, observational (interventional) (case series)	12.0 (5.3–12.0) months	Empagliflozin as add on therapy	Changes in median eGFR, median HbA1c from baseline to end of follow-up	Urinary tract infection, side effects	Median eGFR remained stable, median HbA1c decreased	Timely publication, real-world study	Descriptive analysis, small sample size
Shah *et al*. 2019 [80]	*N* = 24; prospective, observational (interventional)	6 months	Canagliflozin	Not specified	Body weight, blood pressure, HbA1c, serum creatinine, tacrolimus trough levels	Reduction in weight, blood pressure and HbA1c, tacrolimus trough levels unchanged	Only study with canagliflozin	Descriptive, small sample size, only 1 woman
Sánchez Fructuoso *et al*. 2023 [81]	*N* = 338 (*N* = 204 with PTDM, *N* = 134 with T2DM); multicentre, prospective, observational (interventional)	6 months	Canagliflozin, empagliflozin, dapagliflozin, ertugliflozin	Assess adverse events, especially UTIs and/or mycoses in DKTRs placed on SGLT2i treatment	Haemoglobin, eGFR, UACR and/or UPCR, glycaemia (FPG, HbA1c), lipid metabolism	26% patients had an adverse event over 6 months, the most frequent being a UTI (14% patients). In 10% patients, SGLT2i were suspended (mostly because of UTI). However, in a *post hoc* subgroup analysis, UTIs were similar between DKTRs treated with SGLT2i over 12 months, compared with non-DKTRs (17.9% versus 16.7%). Body weight, blood pressure, fasting glycaemia, HbA1c uric acid, UPCR lower after SGLT2i treatment; magnesium and haemoglobin levels higher	The study provides comprehensive and useful clinical information, due in particular to its adequate sample size. Well designed (in the absence of funding for large RCTs). Meaningful way of researching UTI risk in this context	12-months’ follow-up not yet completed in *N* = 105 patients at the time of publication

Green coloured boxes: randomized controlled trials; yellow-coloured boxes: prospective observational/(interventional) studies.

ns: not statistically significant; SAE: serious adverse events; IMT: intima media thickness; AUC: area under the curve; BMI: body mass index; NODAT: new-onset diabetes after transplantation; SGLT2i: SGLT2 inhibitor; ITT: intention-to-treat; ADPKD: autosomal dominant polycystic kidney disease; DM2: type 2 diabetes mellitus; 2hPG: 2-h plasma glucose; FPG: fasting plasma glucose; sig.: significant; IQR: interquartile range; WHR: waist-to-hip ratio; UTI: urinary tract infection; DKTR: diabetic KTR; UACR: urine albumin:creatinine ratio; UPCR: urine protein:creatinine ratio; PP: per protocol.

**Table 1B:** Retrospective studies on glucose-lowering agents after kidney transplantation or SOT including kidney.

**Study**	**Study size**	**Organ**	**Primary results**	**Secondary results**	**Strength**	**Weakness**
Insulin						
Chandra *et al*. 2023 [82]	*N* = 23 (*N* = 10 treated with insulin isophane, *N* = 13 treated with insulin glargine)	Kidney	12 episodes of hypoglycaemia in glargine-treated PTDM patients compared with 3 in isophane-treated PTDM patients (*P *= .056)	Significantly lower blood glucose and HbA1c in the glargine vs. isophane group. In the glargine group, 8 out of 12 hypoglycaemic episodes were nocturnal (1 out of 3 hypoglycaemic episodes were nocturnal in the isophane group)	First report on hypoglycaemia risk with various basal insulin regimens	Patient population predominantly male (87% males) and of relatively young age (average age <40 years in both groups)
Sulfonylureas						
Tuerk *et al*. 2008 [83]	*N* = 47 gliquidone + 28 rosiglitazone (*N* = 75)	Kidney	Mean fasting blood glucose improved, success rate was similar in both groups	In 4 patients the dose of gliquidone therapy had to be reduced due to hypoglycaemia. Pretreatment with other antidiabetics was identified as a negative prognostic factor	First report on SUs in PTDM	Comparison against TZDs (rosiglitazone) with non-standard treatment goals may be somewhat unusual
Metformin						
Kurian *et al*. 2008 [84]	*N* = 32 in the metformin and *N* = 46 in the thiazolidinedione group (pioglitazone, rosiglitazone)	Kidney	No significant difference in HbA1c before and after metformin therapy or thiazolidinedione therapy	No case of lactic acidosis in the metformin group. A slight decrease in eGFR was only significant in the preexisting DM group	Long observational period, first data on safety of metformin	The fact that no treatment effect was observed may not be meaningful in view of sample size and study design
Stephen *et al*. 2014 [60]	*N* = 46 914 (4609 with metformin, 42 305 non-metformin glucose-lowering agent	Kidney	Metformin claims were filled later and were associated with higher eGFR before the first claim	Metformin was associated with lower adjusted hazard for living and deceased donor allograft survival at 3 years. Metformin was associated with lower mortality	Sample size, outcome data	No clear distinction between DM and PTDM, bias by indication
Kwon *et al*. 2023 [59]	*N* = 1193 with metformin, *N* = 802 without	Kidney	Metformin reduced death-censored graft failure, no association with all-cause mortality	No association with BPAR, no confirmed case of lactic acidosis	Sample size, outcome data	Bias by indication
Thiazolidinediones						
Pietruck *et al*. 2005 [85]	*N* = 22 (rosiglitazone)	Kidney	73% had sufficient glycaemic control		Diabetologically comprehensive. Novelty at that time. Duration of follow-up	Sample size
Luther and Baldwin 2004 [86]	*N* = 10 with DM2 and PTDM in KTR and LTR (pioglitazone)	Kidney	Mean HbA1c and mean total daily insulin dose was significantly lower after pioglitazone initiation. Mean serum creatinine levels did not change. Mean blood tacrolimus levels were lower in the pioglitazone group (no difference in dose-normalized tacrolimus blood levels)	Mean BMI increased after pioglitazone. Mean daily prednisolone dose decreased non- significantly. No significant fluid retention and no differences in mean serum lipid values after pioglitazone initiation	Emphasis on safety. Duration of follow-up	Study design itself, potential selection bias, sample size, similarity to study by Baldwin and Duffin
Kurian *et al*. 2008 [84]	*N* = 32 in the metformin and *N* = 46 in the thiazolidinedione group (pioglitazone, rosiglitazone)	Kidney	No significant difference in HbA1c before and after metformin therapy or thiazolidinedione therapy	No case of lactic acidosis in the metformin group. A slight decrease in eGFR was only significant in the preexisting DM group	Long observational period, first data on safety of metformin	The fact that no treatment effect was observed may not be meaningful in view of sample size and study design
Meglitinides						
Türk *et al*. 2006 [87]	*N* = 44 (*N* = 23 repaglinide, *N* = 21 rosiglitazone)	Kidney	After 6 months, 14/23 patients showed successful repaglinide treatment (significant improvement of blood glucose concentrations and HbA1c <7%, no other medication needed)	No significant change in creatinine, cyclosporine A and tacrolimus levels. Similar success rate and HbA1c as in rosiglitazone group	First report on glinides in PTDM	Comparison of various subgroups with non-standard treatment goals
GLP-1 receptor agonists						
Liou *et al*. 2018 [88]	*N* = 7 (liraglutide)	Kidney	Glycaemia improved incl. HbA1c	eGFR improved	Long treatment duration	Small sample size
Singh *et al*. 2019 [89]	*N* = 63 (dulaglutide)	Kidney, liver, heart	Weight loss	Reduction in insulin requirements	Relatively large cohort	Inhomogeneous cohort (multiple organs)
Thangavelu *et al.* 2020 [90]	*N* = 19	Kidney, liver, heart	Stability of the tacrolimus level	Reduction in body weight, BMI and HbA1c	Relatively early study	Inhomogeneous cohort (multiple organs), small sample size
Singh *et al*. 2020 [91]	*N* = 63 (dulaglutide) *N* = 25 (liraglutide	Kidney, liver, heart	Weight loss	Reduction in insulin requirement	Relatively large cohort	Similar data as previous study
Vigara *et al*. 2022 [92]	*N* = 50 (semaglutide, liraglutide, duaglutide)	Kidney	Improvement in eGFR and reduction in proteinuria	Body weight reduction, improvement in HbA1c	Relatively large cohort	Exclusion criteria not clear
Sweiss *et al*. 2022 [93]	*N* = 118, 70% KTRs, 32% PTDM (liraglutide, dulaglutide, semaglutide, exenatide)	Kidney, lung, liver	Significant difference fasting blood glucose and HbA1c at baseline to 3- to 12-month nadir, weight loss	7% nausea, 4% pancreatitis, 7% hypo- glycaemic events	Large cohort of SOT with GLP-1-RA treatment	Various transplanted organs and various GLP-1-RA
DPP4 inhibitors						
Sanyal *et al*. 2013 [94]	*N* = 21 (linagliptin)	Kidney	Linaglitpin monotherapy was effective for glycaemic control in patients with NODAT	Insulin requirement in 2 patients, 1 hypoglycaemic episode	Early real-world data	Entirely descriptive
Boerner *et al*. 2014 [95]	*N* = 22 (sitagliptin)	Kidney	Diabetes control (defined by HbA1c) improved at 6 months and persisted at 12 months	Graft function (serum creatinine and eGFR) did not differ at month 12. No effect on liver transaminase levels and rare occurrence of transplant associated adverse events	Systematic follow-up	Entirely descriptive
Bae *et al*. 2016 [96]	*N* = 65 (vildagliptin, sitagliptin, linagliptin)	Kidney	HbA1c at 3 months significantly decreased from baseline in the linagliptin group compared with other DPP4i	Cyclosporin trough levels were increased in the sitagliptin group compared with the vildagliptin group	Various DPP4 inhibitors analysed	Superiority of one gliptine versus others is clinically implausible and not known in DM2, may have been dose-dependent
Guardado-Mednoza *et al*. 2019 [97]	*N* = 14 (linagliptin + basal (NPH) and lispro insulin) *N* = 14 basal (NPH) and lispro insulin	Kidney	Significant lower fasting plasma glucose levels in the linagliptin plus insulin group after 5 days and at 1 year	Lower insulin doses in the insulin plus linagliptin group and less severe hypoglycaemic events	Data from the early post-transplant period	Treatment duration unclear, therefore, follow-up data not meaningful
Sanyal *et al*. 2021 [98]	*N* = 95 any agent [all received linagliptin (alone or in combination)]	Kidney	NODAT patients achieved long-term glycaemic control and improved renal function	Most patients needed a combination therapy. Linagliptin was effective without producing hypoglycaemia	Manuscript describes a real-world outpatient scenario	Bias by indication
SGLT2is						
Rajasekeran *et al.* 2017 [99]	*N* = 10 (6 KTRs, 4 SPKTs, PTDM and T2DM) (canagliflozin)	Kidney	Meaningful changes in various parameters (incl. HbA1c, weight, and blood pressure), but none of them significant		First study of SGLT2is in transplanted patients	Small sample size
Attallah and Yassine 2019 [100]	*N* = 8 (empagliflozin)	Kidney	Increase in creatinine, decrease in HbA1c, body weight and urinary protein excretion		Meaningful HbA1c reduction shown for patients with excellent allograft function	Descriptive, small sample size
AlKindi *et al*. 2020 [101]	*N* = 8 (empagliflozin, dapagliflozin)	Kidney	Decrease in HbA1c and body mass index, kidney function remained stable		Meaningful HbA1c reduction shown for patients with excellent allograft function	Descriptive, small sample size
Song *et al*. 2021 [102]	*N* = 50 (empagliflozin, canagliflozin, dapagliflozin)	Kidney	Weight reduction	Improvement in hypomagnesemia, reduction in insulin requirement	Relatively large cohort	Low incidence of UTIs is difficult to interpret (more clarity would have been helpful)
Lim *et al*. 2022 [103]	*N* = 226 (empagliflozin, dapagliflozin) among *N* = 2083 (propensity score matching 1:3)	Kidney	Improvement in a composite outcome, consisting of all-cause mortality, death-censored graft failure, and serum creatinine doubling	Graft failure reduced (this item was also part of the composite outcome)	First study to describe hard outcome data in KTRs	Written like an RCT (misleading)
Lemke *et al*. 2022 [104]	*N* = 39 (canagliflozin, dapagliflozin)	Kidney	Decrease in HbA1c	Kidney function and tacrolimus levels not meaningfully altered	Honest discussion of therapy pros and cons	UTIs not clarified further

Both tables contain studies from patients with disorders of the glucose metabolism that became known after transplantation (hyperglycaemia/PTDM/IGT). If studies were entirely conducted with patients who had type 2 diabetes before transplantation, they were not listed.

DM: diabetes mellitus; DM2: type 2 diabetes mellitus; NODAT: new-onset diabetes after transplantation; BMI: body mass index; GLP-1-RA: GLP-1 receptor agonist; DPP4i: DPP4 inhibitor; SU: sulfonylurea; TZDs: thiazolidinediones; BPAR: biopsy proved acute rejection.

Metformin is cheap and easily available. While advised for use only with estimated glomerular filtration rate (eGFR) ≥30 mL/min/1.73 m^2^, renal restrictions are not an absolute requirement [58]. Observational studies show an association with lower risk for death-censored graft failure [59] and post-transplant mortality [60, 61] but not cardiovascular-related mortality. Metformin may be an appropriate choice for solid organ transplant recipients at low risk for adverse cardio-renal outcomes or if access to novel anti-diabetics is an issue. However, for solid organ transplant recipients at moderate to high risk for adverse cardio-renal outcomes with no accessibility issues, the consensus opinion was novel anti-diabetic therapies should be strongly considered before metformin.

SGLT2 inhibitors can be used for the treatment of PTDM once stable graft function is achieved [62]. Initiation should be influenced by comorbidities like heart failure (supporting use) and significant urosepsis or severe mycotic genital infection risk (discouraging use), although current studies have not shown increased urinary tract infection risk with SGLT2 inhibitors (see Tables 1A and 1B). Enthusiasm for early post-operative commencement will be influenced by local urological practices (e.g. length of post-operative urinary catheter placement, ureteric stent removal). Improvement of glycaemic control may vary based on kidney function (less effective at lower eGFR) [62]. Awareness of the risk for euglycaemic diabetic ketoacidosis is critical, especially in patients with insulin deficiency. SGLT2 inhibitors should be suspended if fasting is required or during an acute illness.

GLP-1 receptor agonists are preferable in patients with obesity. Several non-randomized published reports indicate an acceptable safety profile with no increased rejection or graft failure risk, although gastrointestinal side effects are common. Appropriate education is required for patients who are initiated on incretin mimetics with emphasis on slow dose up-titration to improve tolerance, and suspension of treatment with acute illness [25].

Insulin should be used for treatment of post-operative hyperglycaemia. For stable patients, oral or non-insulin injectable agents (and their combination) are preferable unless diabetes control cannot be achieved. Of note, data on the glucose-lowering effect of basal insulin in KTRs exist for basal neutral protamine Hagedorn (NPH)-insulin alone [48], the peak effect of which can be matched to the glucose peak exhibited by KTRs in the afternoon.

Dipeptidylpeptidase 4 (DPP4) inhibitors are safe but demonstrate no cardio-renal benefit. Thiazolidinediones are better options than sulfonylureas and meglitinides (both have risk of hypoglycaemia), and no evidence exists for alpha-glucosidase inhibitors. Meeting participants agree these drug classes have the lowest priority for clinical use.

In summary, and in view of the pros and cons for each pharmacological therapy, meeting participants agreed that any decision to initiate one glucose-lowering agent versus another is best guided by a patient-dependent hierarchy (shown in Fig. 2) if accessibility is not an issue. Personalization of glucose-lowering therapy is essential, with treatment goals depending on comorbidities, awareness of hypoglycaemia risk and allograft function.

## OPINION STATEMENT 8: INCREASE COLLABORATIVE RESEARCH BETWEEN ACADEMIC MEDICINE, MULTI-DISCIPLINARY CLINICAL TEAMS, INDUSTRY PARTNERS AND PATIENTS

Exclusion of SOT recipients from pioneering cardiovascular and renal outcome trials of new glucose-lowering agents has resulted in sub-optimal uptake post-transplantation. Observational studies and RCTs relating to PTDM are in progress (see Supplementary data, Table S3), but more are required and should target at-risk groups for maximum benefit. Patient-reported outcomes, health economic analyses and cost effectiveness models are lacking and require dedicated studies and incorporation as secondary outcomes into RCTs where feasible (suggested PTDM clinical trial endpoints in Supplementary data, Table S4). Lack of robust PTDM data capture by national transplant registries limits the ability to ascertain PTDM-associated outcomes [63]. Acquiring these data should be encouraged to improve our understanding of long-term outcomes with record linkage. Collaboration between healthcare professionals, academic groups, industry and patient groups is essential.

Finally, most published research is after kidney transplantation, but PTDM is a complication affecting all SOT recipients with prevalence rates between 20% and 40% in heart, lung and liver transplant recipients [64]. In a Danish SOT cohort (*n* = 959), the highest incidence of PTDM is seen 46–365 days post-transplantation. SOT recipients with PTDM had higher risk for all-cause mortality (1.89, 95% CI 1.17–3.06), with cardiovascular and cancer-related causality more common than in non-diabetic SOT recipients [18]. More studies are warranted in non-renal transplant cohorts. While most of this report is valid across SOT cohorts, bespoke differences may be apparent between different solid organ settings to justify organ-specific versus organ-generic recommendations.

## CONCLUSION

PTDM is a complex and multi-factorial post-transplant complication, spanning a continuum of disease that may begin prior to transplantation in many cases. This Meeting Report summarizes proceedings from the 3rd International PTDM Consensus meeting, reflecting expert opinion. Optimizing long-term outcomes after SOT, with attenuation of both premature mortality and/or graft loss, is a clinical priority. Therefore, improving our diagnosis, prevention and management of PTDM should be considered an integral component of long-term post-transplant care.

## Supplementary Material

gfad258_Supplemental_Files

## Data Availability

No empirical data collected for this manuscript.
