# Supplementary material for: International consensus on post-transplantation diabetes mellitus
Source: Nephrol Dial Transplant. 2024 Jan 3;39(3):531–49. doi: 10.1093/ndt/gfad258 (PMC11024828; doi:10.1093/ndt/gfad258)
Supplement: gfad258_Supplemental_Files [file gfad258_Supplemental_Files.zip › Table S1.docx]

**Table S1. Measures of glycemia after organ transplantation**

| **Test** | **Diagnosis** | **ADA criteria^3^** | **Comment** |
| --- | --- | --- | --- |
| *RPG* | PTDM | Classic symptoms of hyperglycemia or hyperglycemic crisis AND RPG ≥200 mg/dL (11.1 mmol/L) |  |
| *FPG* | PTDM | FPG ≥126 mg/dL (7.0 mmol/L) | Often poor overlap with 2hPG. |
|  | IFG | FPG 100–125 mg/dL (5.6–6.9 mmol/L) |  |
|  | Normal fasting glucose | FPG <100 mg/dL (5.6 mmol/L) |  |
| *OGTT* | PTDM | 2hPG ≥200 mg/dL (11.1 mmol/L) **or** FPG ≥126 mg/dL (7.0 mmol/L) | Most sensitive test, strong association with outcomes  necessary to detect IGT. |
|  | Prediabetes |  |  |
|  | IGT | 2hPG 140–199 mg/dL (7.8–11.0 mmol/L) AND FPG <126 mg/dL (7.0 mmol/L) |  |
|  | IFG | FPG 100–125 mg/dL (5.6–6.9 mmol/L) AND 2hPG <200 mg/dL (11.1 mmol/L) |  |
|  | NGT | FPG <100 mg/dL (5.6 mmol/L) AND 2hPG <140 mg/dL (7.8 mmol/L) |  |
| *HbA1c* | PTDM | HbA1c ≥6.5% (48 mmol/mol) | Often poor overlap with 2hPG and FPG. Weak association with outcomes, a normal result does not exclude PTDM or prediabetes. |
|  | Increased risk for diabetes | HbA1c 5.7–6.4% (39–47 mmol/mol) |  |
|  | Normal | HbA1c <5.7% (39 mmol/mol) |  |

Legend: ADA = American Diabetes Association, RPG = random plasma glucose, FPG = fasting plasma glucose, 2hPG = OGTT derived 2-hour plasma glucose, OGTT = oral glucose tolerance test, HbA1c = glycated hemoglobin A1c, PTDM = post transplantation diabetes mellitus, IFG = impaired fasting glucose, IGT = impaired glucose tolerance, NGT = normal glucose tolerance
