# Supplementary material for: International consensus on post-transplantation diabetes mellitus
Source: Nephrol Dial Transplant. 2024 Jan 3;39(3):531–49. doi: 10.1093/ndt/gfad258 (PMC11024828; doi:10.1093/ndt/gfad258)
Supplement: gfad258_Supplemental_Files [file gfad258_Supplemental_Files.zip › Table S2.docx]

**Table S2.** Published studies exploring diagnosis of PTDM.

| **Study** | **Time** | **Timepoint post Tx** | **Nr of OGTTs** | **Design and participants** | **Analyses** | **Reference test** | **TPR (Exact 95% CI)** | **TNR (Exact 95% CI)** | **Strength** | **Weakness / differences to other studies** |
| --- | --- | --- | --- | --- | --- | --- | --- | --- | --- | --- |
| **HBA1C 6.5% (48 mmol/mol) VERSUS OGTT FOR PTDM DIAGNOSIS** | | | | | | | | | | |
| Kurnikowski et al, 2022 (1) | 2012-2018 | 6 months | 188 | Retrospective analysis in participants of a multicenter randomized clinical trial. | HbA1c and FPG versus OGTT for PTDM and prediabetes. Combined criteria. Longitudinal PTDM development. | OGTT (2hPG and/or FPG) (primary analysis: 2hPG) | 0.21 (0.06, 0.46) | 0.98 (0.95, 1.00) | **General:** Three timepoints in the same participants. Multicenter study.  **Selection:** Consecutive, no case control design, no inappropriate exclusions. Detailed report of inclusion/exclusions.  **Index test:** Prespecified thresholds, same visit as OGTT  **Reference test:** OGTT  **Flow:** Prespecified test timepoint in all patients. | **Selection:** Participants fulfilling inclusion criteria of RCT. Only FPG for pretransplant screening.  **Index test**: -  **Reference test:** 2hPG primary reference (2hPG+FPG reported in supplement), no confirmation OGTT.  **Flow:** - |
|  |  | 12 months | 183 |  |  |  | 0.50 (0.25, 0.75) | 0.97 (0.93, 0.99) |  |  |
|  |  | 24 months | 163 |  |  |  | 0.58 (0.33, 0.80) | 0.98 (0.94, 1.00) |  |  |
| Ussif et al, 2019 (2) | 2012-2016 | 12 months | 494 | Observational study in central transplant center of Norway where patients routinely receive an OGTT at 1 year | HbA1c and combined HbA1 and FPG vs OGTT | OGTT (2hPG and/or FPG) | 0.43 | 0.97 | **Selection:** No case control design, no inappropriate exclusions. Detailed report of flow/inclusions. Pretransplant OGTT for DM exclusion.  **Index test:** Prespecified threshold. Same visit as OGTT.  **Reference test:** OGTT  **Flow:** Prespecified test timepoint in all patients. | **Selection: -**  **Index test:** -  **Reference test:** No confirmation OGTT.  **Flow:** - |
| Pimentel et al, 2015 (3) | 2012 - 2014 | 4 months | 122 | Prospective observational study in in single center cohort | HbA1c versus OGTT for PTDM  Stratification for OGTT using HbA1c. | OGTT (2hPG and/or FPG) | 0.44 (0.26, 0.62) | 0.98 (0.92, 1.00) | **Selection:** Consecutive, no case control design. excl. of anemia/influencing factors**.**  **Index test:** Prespecified threshold. Same visit as OGTT.  **Reference test:** OGTT  **Flow:** Prespecified test timepoint in all patients. | **Selection**: Only FPG for pretransplant screening  **Index test**: -  **Reference test**: No confirmation OGTT.  **Flow**: - |
| Clayton et al, 2015 (4) | 2005-2013 | 3 months | 119 | Retrospective observational study in in single center cohort | HbA1c versus OGTT for PTDM | OGTT (2hPG) | 0.20 (0.08, 0.37) | 0.94 (0.87, 0.98) | **Selection:** No case control design.  **Index test:** Prespecified threshold  **Reference test:** OGTT  **Flow:** - | **General**: Letter, limited reporting, retrospective  **Selection:** Immunosuppression differs (mostly tacrolimus, also CysA and Everolimus or Sirolimus), pretransplant DM screening?  **Index test:** -  **Reference test:** 2hPG reference only. no confirmation OGTT.  **Flow:** HbA1c measurement between 2 weeks before and 6 weeks after the OGTT. only 40% of performed OGTTs also had HbA1c measurements. |
| Yates et al, 2013 (5) | 2010-2012 | 6 weeks | 26 | Prospective observational study in single center cohort | Afternoon capillary blood glucose, FPG, HbA1c, OGTT versus PTDM (any criteria)  HbA1c vs OGTT |  | Not reported | Not reported | **General:** Three timepoints.  **Selection:** Consecutive, no case control design, no inappropriate exclusions.  **Index test:** Same visit as OGTT  **Reference test:** OGTT  **Flow:** Prespecified test timepoints | **General:** small cohort, only numbers given, no sens./spec. or 95%CI reported. OGTTs at 3 and 12 months were from different participants.  **Selection:** Excl. patients with planned pregnancy. Only FPG for pretransplant screening.  **Index test**: -  **Reference test:** no confirmation OGTT.  **Flow:** 6 weeks OGTT only in patients with high steroid doses. accuracy evaluated only in month 3 and 12 combined. |
|  |  | 3 months | 50 |  |  | OGTT (2hPG and/or FPG) | 0.43 (0.18, 0.71)  (combined month 3 and 12) | 0.95 (0.89, 0.99)  (combined month 3 and 12) |  |  |
|  |  | 12 months | 51 |  |  |  |  |  |  |  |
| Rosettenstein et al, 2015 (6) | 2008 - 2011 | 3 months | 83 | Prospective observational study in single center cohort | Average random blood glucose, HbA1c fructosamine or HOMA-Insulin Resistance versus IFG/IGT, PTDM, IGT/PTDM | OGTT (2hPG and/or FPG) | 71.4 (0.42, 0.92) | 95.7 (0.88, 0.99) | **Selection:** Consecutive, no case control.  **Index test:** Prespecified threshold  **Reference test:** OGTT  **Flow:** Prespecified test timepoint in all patients. | **General:** small cohort  **Selection**: Only FBG and RBG for pretransplant screening. Immunosuppression differs (Tacrolimus in most patients, or CysA)  **Index test**: -  **Reference test**: no confirmation OGTT.  **Flow**: Flow and missing values not reported. |
| Eide et al, 2015 (7) | 1999-2011 | 10 weeks | 1544 | Observational study in central transplant center of Norway where patients routinely receive an OGTT at 1 year | HbA1c versus combined FPG and OGTT screening  FPG, 2hPG and combined FPG and HbA1c criteria as index tests  Additional analysis in patients with normal FPG | Repeatedly diabetic FPG or OGTT (2hPG and/or FPG) | 0.38 | 0.86 | **Selection:** No case control design, no inappropriate exclusions. Pretransplant OGTT for DM exclusion. Additional analysis in patients without anemia.  **Index test:** Prespecified thresholds. Same visit as OGTT.  **Reference test:** OGTT  **Flow:** Prespecified test timepoint in all patients. | **Selection:** Varying immunosuppression over time (tacrolimus/cyclosporine), underage individuals included (16-18y), overlap w. previously published cohort (Valderhaug et al).  **Index test:** -  **Reference test:** No isolated analysis of OGTT alone as reference test for comparison HbA1c versus OGTT. No confirmation OGTT.  **Flow:** Nr. of performed and missing HbA1c values? Nr. of patients in 2x2 table? |
| Shabir et al, 2013 (8) | 2009-2010 | 3 months | ?  (71 recruited) | Prospective observational study in single center cohort | HbA1c vs 2hPG  HbA1c vs FPG  (ROC curve analysis) optimum cut-off HbA1c value | OGTT | HbA1c 6.5% not reported | HbA1c 6.5% not reported | **Selection:** Consecutive, no case control design, no inappropriate exclusions. Two timepoints evaluated.  **Index test:** Same visit as OGTT  **Reference test:** OGTT  **Flow:** Prespecified test timepoints | **General:** Small cohort, patient flow, pos. and neg. results not provided. Sensitivities are higher than in all other studies.  **Selection:** Only FPG for pretransplant screening. Also minors included (at least one 17 y. old)  **Index test**: HbA1c 6.5% not provided, only ROC curve analysis.  **Reference test:** 2hPG and FPG separately reported but not combined. No confirmation OGTT  **Flow**: Nr. of performed and missing HbA1c values? Nr. of patients in 2x2 table? |
|  |  | 12 months | ? |  |  |  | HbA1c 6.5% not reported | HbA1c 6.5% not reported |  |  |
| Valderhaug et al, 2009  (9) | 1995 -2006 | 10 weeks | 929 | Observational study in central transplant center of Norway where patients routinely receive an OGTT at 1 year | FPG and HbA1c thresholds for OGTT referral | OGTT (2hPG in patients with normal FPG) | HbA1c 6.5% not reported | HbA1c 6.5% not reported | **Selection:** consecutive, no case control design. Detailed report of inclusion/exclusions, pretransplant OGTT for DM exclusion.  **Index test:** same visit as OGTT.  **Reference test:** OGTT  **Flow:** Prespecified test timepoint in all patients. | **General:** No specificity reported  **Selection:** Varying immunosuppression over time (cyclosporine not tacrolimus in most)  **Index test:** no prespecified HbA1c thresholds. Only cutoffs below 6.0 reported.  **Reference test**: 2hPG but FPG <126 mg/dL. Plasma glucose was calculated from venous whole blood glucose. (relevance?), no confirmation test.  **Flow:** Participants with FPG ≥126 mg/dL were excluded from evaluation of HbA1c, although OGTTs were available. |

**Legend**: TPR: true positive rate, TNR: true negative rate. 95%CI: 95% confidence interval. PTDM Post transplant diabetes mellitus. FPG: fasting plasma glucose, 2hPG: 2-hour plasma glucose. OGTT: oral glucose tolerance test. HbA1c: glycated hemoglobin A1c. Reference test/TPR/TNR: refer to diagnostic accuracy of HbA1c 6.5% versus OGTT for PTDM diagnosis. Where available, the 2hPG and/or FPG OGTT reference was reported instead of 2hPG only.

**General disadvantages of the diagnostic accuracy studies:**

There are methodological differences and lack of reporting is some aspects of the published studies (see Table 1). Pimentel et al, however, evaluated risk of bias and concerns regarding applicability and they found low risk in all but 2/6 studies, where patient selection and/or index test posed unknown risk of bias (10). Diagnostic accuracy studies for PTDM are observational studies that evaluate tests accuracy cross-sectionally. Most important limitations in these studies are: Few of these studies have an adequate number of patients for the evaluation of test sensitivity versus OGTT. There are only few nationwide cohorts and most studies are single center studies. The choice and dose of immunosuppression is heterogenous, especially over time. Most studies have been performed in Europe and there Is lack in evidence in non-White ethnic groups. Only one study evaluated sex/gender differences as a secondary analysis. Absolute numbers, 2x2 Tables and 95%CI or missing values are often not reported. Most studies/centers do not perform confirmation of OGTT diagnosis which could lead to an overestimation of PTDM cases, although the impact of this practice is unknown. There are longitudinal outcome studies using different diagnostic criteria but no study evaluated improvement of outcomes.

No evidence/research gap:

- **PTDM diagnosis using OGTT screening versus FPG/HbA1c improves outcomes post-transplant**
- **IGT diagnosis using OGTT screening versus FPG/HbA1c improves outcomes post-transplant**
- **Repeated OGTT screening improves outcomes post-transplant**
- **Stratification for posttransplant OGTT referral is effective using diabetes risk factors and prediabetes (IGT/IFG)**

**References**

1. Kurnikowski A, Nordheim E, Schwaiger E, Krenn S, Harreiter J, Kautzky-Willer A et al. Criteria for prediabetes and posttransplant diabetes mellitus after kidney transplantation: A 2-year diagnostic accuracy study of participants from a randomized controlled trial. Am J Transplant 2022.

2. Ussif AM, Åsberg A, Halden TAS, Nordheim E, Hartmann A, Jenssen T. Validation of diagnostic utility of fasting plasma glucose and HbA1c in stable renal transplant recipients one year after transplantation. BMC Nephrol 2019;20(1):12.

3. Pimentel AL, Carvalho LS, Marques SS, Franco RF, Silveiro SP, Manfro RC et al. Role of glycated hemoglobin in the screening and diagnosis of posttransplantation diabetes mellitus after renal transplantation: A diagnostic accuracy study. Clin Chim Acta 2015;445:48-53.

4. Clayton PA, Aouad L, Wyburn KR, Eris JM, Chadban SJ. HbA1c Is Insensitive at Month 3 After Kidney Transplantation. Transplantation 2015;99(5).

5. Yates CJ, Fourlanos S, Colman PG, Cohney SJ. Screening for new-onset diabetes after kidney transplantation: limitations of fasting glucose and advantages of afternoon glucose and glycated hemoglobin. Transplantation 2013;96(8):726-731.

6. Rosettenstein K, Viecelli A, Yong K, Nguyen HD, Chakera A, Chan D et al. Diagnostic Accuracies of Glycated Hemoglobin, Fructosamine, and Homeostasis Model Assessment of Insulin Resistance in Predicting Impaired Fasting Glucose, Impaired Glucose Tolerance, or New Onset Diabetes After Transplantation. Transplantation 2016;100(7).

7. Eide IA, Halden TA, Hartmann A, Åsberg A, Dahle DO, Reisæter AV et al. Limitations of hemoglobin A1c for the diagnosis of posttransplant diabetes mellitus. Transplantation 2015;99(3):629-635.

8. Shabir S, Jham S, Harper L, Ball S, Borrows R, Sharif A. Validity of glycated haemoglobin to diagnose new onset diabetes after transplantation. Transpl Int 2013;26(3):315-321.

9. Valderhaug TG, Jenssen T, Hartmann A, Midtvedt K, Holdaas H, Reisaeter AV et al. Fasting plasma glucose and glycosylated hemoglobin in the screening for diabetes mellitus after renal transplantation. Transplantation 2009;88(3):429-434.

10. Pimentel AL, Cavagnolli G, Camargo JL. Diagnostic accuracy of glycated hemoglobin for post-transplantation diabetes mellitus after kidney transplantation: systematic review and meta-analysis. Nephrol Dial Transplant 2017;32(3):565-572.
