# Supplementary material for: International consensus on post-transplantation diabetes mellitus
Source: Nephrol Dial Transplant. 2024 Jan 3;39(3):531–49. doi: 10.1093/ndt/gfad258 (PMC11024828; doi:10.1093/ndt/gfad258)
Supplement: gfad258_Supplemental_Files [file gfad258_Supplemental_Files.zip › Table S3.docx]

**Table S3. Current recent studies registered on clinicaltrials.gov related to Post-Transplant Diabetes Mellitus**

| **Clinicaltrials.org**  **identifier** | **Study completion** | **Title of project** | **Study narrative** |
| --- | --- | --- | --- |
| Active |  |  |  |
| NCT05702931 | Active, not recruiting | Semaglutide Treatment for Hyperglycemia After Renal Transplantation (Sema-RTx) | Double-blind, placebo-controlled RCT comparing safety and efficacy of oral semaglutide after kidney transplantation |
| NCT04382157 | Recruiting | Magnesium Replacement and Hyperglycemia After Kidney Transplantation | RCT comparing oral magnesium supplementation vs placebo to improve glycemic parameters as measured by an OGTT |
| NCT02849899 | Recruiting | Prevention of Diabetes After Transplantation by Vildagliptin in the Early Post-transplant Period (PRODIG) | Open label RCT comparing early treatment with Vildagliptin to placebo |
| NCT04965935 | Recruiting | Efficacy, Mechanisms and Safety of SGLT2 Inhibitors in Kidney Transplant Recipients  (INFINITI2019) | Double-blind RCT comparing treatment with Dapagliflozin to placebo in KTR with pre-existing pre-existing type diabetes (T2D) or PTDM |
| NCT05240274 | Active, not recruiting | The POWERED Study: Prophylaxis With Metformin to Prevent PTDM | Open label RCT comparing early treatment with Metformin to placebo to prevent PTDM in patients without pre-existing diabetes mellitus |
| NCT05396898 | Recruited, study in progress | Tacrolimus Formulation and Glucose Metabolism After Kidney Transplantation (TAGLUMET Trial) | Randomized cross-over conversion trial to assess glucose metabolism under LCP-tacrolimus vs twice-daily tacrolimus |
| Completed |  |  |  |
| NCT04489043 | October 2021 | Exercise, Prediabetes and Diabetes After Renal Transplantation (EXPRED) | Single group interventional study that evaluates if exercise can prevent PTDM in KTR with proven prediabetes |
| NCT04030013 | July 2019 | Outcome of Diabetes Education Among Renal Transplant Recipients With New Onset Diabetes After Transplantation | RCT of 3 arms: Group education or one to one education for 2 years and control group without structured education. |
| NCT03507829 | May 2018 | Insulin Therapy for the Prevention of NODAT Prospective Study in Non-Diabetic De Novo Kidney Transplant Recipients (ITP-NODAT) | Open label RCT comparing aggressive glycemic control with early institution of insulin therapy to standard of care |
| NCT01680185 | May 2018 | Sensor-Augmented Insulin-Pump Therapy in New-onset Diabetes After Transplantation (SAPT-NODAT) | Open label study of intensive subcutaneous insulin treatment with short acting insulin, applied continuously through an insulin pump in comparison to a standard of care control group |
| NCT03642184 | December 2020 | Efficacy and Safety of Empagliflozin in  NODAT | Open label RCT testing of empagliflozin versus linaligliptin for treatment of NODAT |
| NCT03113110 | May 2018 | Empagliflozin in Post-Transplantation Diabetes Mellitus | Single group interventional study that evaluates the use of Empagliflozin for PTDM patients on previous antidiabetic treatment |
| NCT01928199 | October 2020 | Efficacy Study of Sitagliptin to Prevent New-onset Diabetes After Kidney Transplant | Open label RCT to evaluate the efficacy of sitagliptin to prevent the development of NODAT in previously non-diabetic patients with post-operative hyperglycemia following kidney transplant |
| NCT03961256 | June 2021 | A Phase 2, Prospective, Randomized, Multicenter, Open-Label, Controlled  Trial to Assess the Efficacy and Safety of Exenatide SR for the Prevention of  Diabetes After Kidney Transplantation | Open label RCT to determine if Exenatide SR, is well tolerated in kidney transplant patients with elevated blood glucose levels, and if it’s effective in preventing  diabetes |

*KTR: kidney transplant recipients; NODAT: New Onset Diabetes After Kidney Transplantation; OGTT: oral glucose tolerance test; PTDM: Post-Transplant Diabetes Mellitus; RCT: Randomized controlled trial.
