# Supplementary material for: International consensus on post-transplantation diabetes mellitus
Source: Nephrol Dial Transplant. 2024 Jan 3;39(3):531–49. doi: 10.1093/ndt/gfad258 (PMC11024828; doi:10.1093/ndt/gfad258)
Supplement: gfad258_Supplemental_Files [file gfad258_Supplemental_Files.zip › Table S4.docx]

**Table S4. Clinical trial endpoints for PTDM related research**

| **Clinical endpoints** | **Surrogate endpoints** |
| --- | --- |
| **Long term** | |
| - Mortality |  |
| - Graft survival | - Delta GFR |
| - 3-point MACE^1^: - non-fatal cardiac infarction - non-fatal stroke - death from cardiovascular disease - MACE+/CVD: - Coronary heart disease (revascularization/cardiac infarction/unstable a.p.) - stroke/transient ischemic attack/ cerebral stenting/revascularization - admission for heart failure - Peripheral vascular disease (severe obstruction, amputation, revascularization) - death from cardiovascular disease |  |
| - Cancer |  |
| - Infections - urinary tract infections - foot ulcers |  |
| - Diabetic nephropathy | - Albuminuria - Renal histology - Gene expression, miRNA - Inflammation |
| - Retinopathy |  |
| **Short term** | |
| - Diabetes control - Remission of metabolic syndrome/PTDM - Progression from pre-diabetes to diabetes | - Glycemic control: - HbA1c - Fasting glucose - Fructosamine - OGTT^2^ - CGM^2^ - Weight loss |
| - Patient reported outcomes |  |

^1^Major adverse cardiovascular event

^2^Oral glucose tolerance test

^3^Continuous glucose monitoring
