# Supplementary figures and images for: International consensus on post-transplantation diabetes mellitus
Source: Nephrol Dial Transplant. 2024 Jan 3;39(3):531–49. doi: 10.1093/ndt/gfad258 (PMC11024828; doi:10.1093/ndt/gfad258)

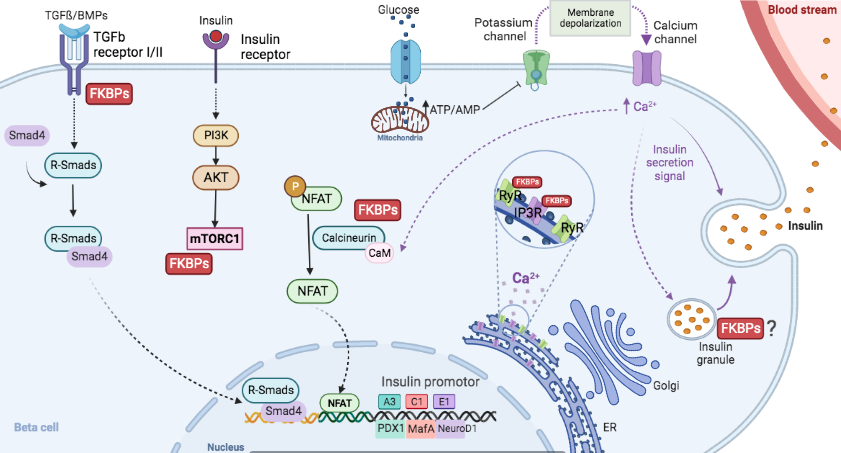

Supplement: gfad258_Supplemental_Files [file gfad258_Supplemental_Files.zip › Figure S2.tiff]

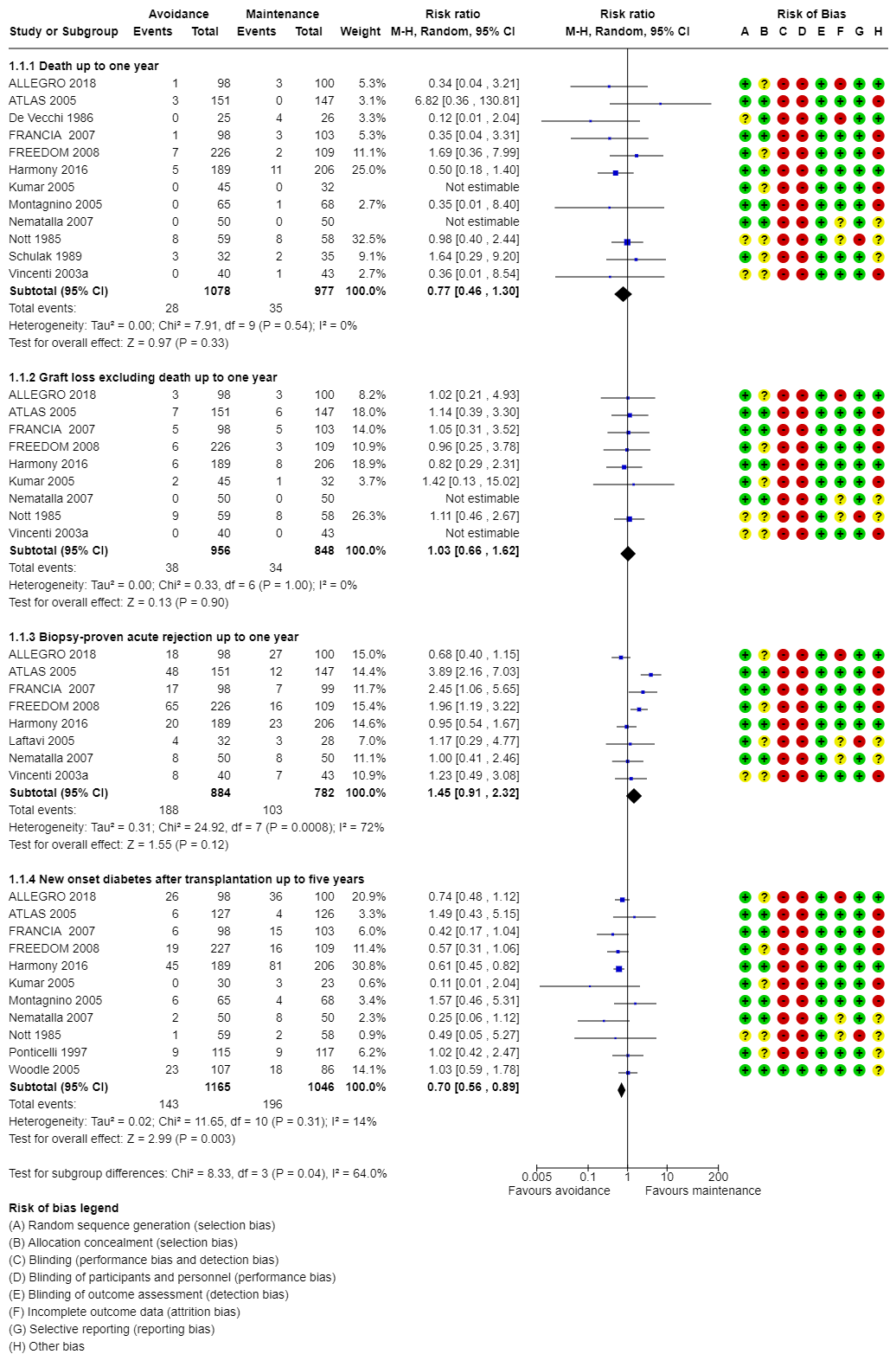

Supplement: gfad258_Supplemental_Files [file gfad258_Supplemental_Files.zip › Figure S3.png]
